# Type I Interferons Promote Germinal Centers Through B Cell Intrinsic Signaling and Dendritic Cell Dependent Th1 and Tfh Cell Lineages

**DOI:** 10.3389/fimmu.2022.932388

**Published:** 2022-07-13

**Authors:** Madelene W. Dahlgren, Adam W. Plumb, Kristoffer Niss, Katharina Lahl, Søren Brunak, Bengt Johansson-Lindbom

**Affiliations:** ^1^ Immunology Section, Lund University, Lund, Sweden; ^2^ Department of Health Technology, Technical University of Denmark, Lyngby, Denmark; ^3^ Novo Nordisk Foundation Center for Protein Research, University of Copenhagen, Copenhagen, Denmark

**Keywords:** Type I Interferons, germinal center (GC) B cells, IgG subclass antibodies, Tfh cells, Th1 cells, antibody responses

## Abstract

Type I interferons (IFNs) are essential for antiviral immunity, appear to represent a key component of mRNA vaccine-adjuvanticity, and correlate with severity of systemic autoimmune disease. Relevant to all, type I IFNs can enhance germinal center (GC) B cell responses but underlying signaling pathways are incompletely understood. Here, we demonstrate that a succinct type I IFN response promotes GC formation and associated IgG subclass distribution primarily through signaling in cDCs and B cells. Type I IFN signaling in cDCs, distinct from cDC1, stimulates development of separable Tfh and Th1 cell subsets. However, Th cell-derived IFN-γ induces T-bet expression and IgG2c isotype switching in B cells prior to this bifurcation and has no evident effects once GCs and *bona fide* Tfh cells developed. This pathway acts in synergy with early B cell-intrinsic type I IFN signaling, which reinforces T-bet expression in B cells and leads to a selective amplification of the IgG2c^+^ GC B cell response. Despite the strong Th1 polarizing effect of type I IFNs, the Tfh cell subset develops into IL-4 producing cells that control the overall magnitude of the GCs and promote generation of IgG1^+^ GC B cells. Thus, type I IFNs act on B cells and cDCs to drive GC formation and to coordinate IgG subclass distribution through divergent Th1 and Tfh cell-dependent pathways.

## Introduction

Neutralizing antibody responses develop within germinal centers (GCs); histological structures that arise within lymphoid tissues due to antigen-driven clonal B cell expansion. GCs are also thought to represent key sites for breach of tolerance in development of autoimmune disease (1). GC B cell responses rely on T follicular helper (Tfh) cells, which support B cell expansion, facilitate antibody affinity maturation and eventually select GC B cells into the compartments of long-lived plasma cells or memory B cells (2).

Type I interferons (IFNs), including a single IFN-β and several IFN-α proteins, are rapidly produced in response to viral and bacterial infections and signal through the common and ubiquitously expressed heterodimeric IFN-α receptor (IFNAR) (3). Initially discovered for their ability to induce the “antiviral state” in host cells, type I IFNs are also associated with a plethora of immune-regulatory functions essential for antiviral immunity (4). The importance of type I IFNs in controlling SARS-CoV-2 is also evident from the presence of neutralizing autoantibodies against these cytokines, or mutations in type I IFN signaling pathways, in a significant proportion of patients developing severe COVID-19 disease (5–7). Furthermore, elevated type I IFN production is a hallmark of systemic autoimmune diseases, with a strong correlation between the IFN gene expression signature and clinical manifestations in SLE (8–11). When co-injected with a protein antigen, IFN-α has sufficient adjuvant activity to induce GC formation (12) and GC B cell responses have been shown to depend on type I IFNs both during viral infections (13) and in autoimmune models (14, 15). While we and others have shown that type I IFN signaling in cDCs augments generation of Tfh cells (16, 17), direct signaling in B- and T- cells has also been implicated in type I IFN-dependent enhancement of humoral immunity and autoimmunity (12, 13, 15, 18, 19). The relative contribution of these pathways, and how they interact with the type I IFN – cDC – Tfh cell axis to enhance and modulate the GC response, is however unclear.

Different IgG subclasses are associated with distinct effector mechanisms. In C57BL/6 mice, complement-fixing IgG2c (IgG2a in the BALB/c strain) is more efficient than other subclasses in neutralizing viruses (20) and is also associated with more severe pathology in lupus models (21). The transcription factor T-bet induces class switch recombination (CSR) to IgG2a/c (22). T-bet expression in B cells appears to have effects beyond CSR (23) and was recently shown to be required for generation of protective anti-influenza stalk region-specific antibodies (24). Mouse IgG2a/c may hence represent a useful surrogate marker for antibody responses driven by T-bet expressing B cells, with relevance for the human setting. Purified IFN-β can enhance production of all IgG subclasses in mice and a similarly broad and type I IFN-dependent isotype distribution is induced through the adjuvant effects of the synthetic dsRNA analogue polyinosinic-polycytidylic acid (poly I:C) (25). Other studies suggest that type I IFNs preferentially promote IgG2a/c (26, 27), and B cell-intrinsic type I IFN signaling mediates IgG2a/c CSR in the T cell-independent response to NP-Ficoll (28). The importance of this pathway in GC B cell responses is however less clear and the IgG2a/c subclass has more frequently been associated with strong Th1 immunity and IFN-γ production (29, 30).

Type I IFNs can both inhibit and promote generation of IFN-γ producing Th1 cells, with a general trend that sustained type I IFN responses during chronic infections suppress Th1 immunity (4). Through binding to endosomal TLR3 and the cytoplasmic RNA helicase MDA5, poly I:C instead triggers a transient systemic type I IFN response that peaks 3-6 hours after injection (31, 32), and an almost identical pattern in type I IFN production is observed for novel mRNA vaccination regimens (33). In contrast to the chronic infection models, this short-lived type I IFN response leads to Th1-biased immunity with abundant IFN-γ production from CD4 and CD8 T cells and in particular from NK cells (31). How this IFN-γ response develops, and what impact it has on GC B cell differentiation remains to be determined.

## Materials and Methods

### Study Design

This study sought to determine pleiotropic effects and identify the cellular targets of acute type I IFN signaling on the germinal center B cell response. By selectively blocking receptor signaling in defined target populations, or permitting signaling to occur only during defined time windows, we unravel how GCs with broad IgG subclass distribution develop in mice immunized with OVA plus poly I:C. All experiments were performed according to protocols approved by the Lund/Malmo animal ethical committee (Sweden).

### Mice

C57Bl/6 mice (wild type [wt]) were purchased from Taconic (Ejby, Denmark), *Ifngr1^-/-^* (B6.129S7-*Ifngr1^tm1agt^*/J) and *Ifng^-/^* ^-^ (B6.129S7-*Ifng^tm1Ts^/J*) mice purchased from The Jackson Laboratory (Bar Harbor, ME, USA). *Il27ra^-/-^* (B6N.129P2-Il27ra^tm1Mak^/J), *Ifnar1^-/-^* (on a C57Bl/6 background), B6.SJL (B6.SJL-Ptprca Pepcb/BoyJ) and OT-II (B6.Cg-Tg(TcraTcrb)425Cbn/J), KN2 (Il4^tm1(CD2)Mmrs^) (34), *CD11c-cre* (B6.Cg-Tg(Itgax-cre)1-1Reiz/J), *XCR1-cre* (Xcr1^Cre-mTFP1^) (35) and *Ifnar1^fl/fl^* (Ifnar1^tm1Uka^) (36) mice were bred and maintained at the Biomedical Center animal facility, Lund University. *Ifnar1^fl/fl^* (Ifnar1^tm1Uka^). CD45.1^+^CD45.2^+^ OT-II and C57Bl/6 mice were generated by breeding B6.SJL (CD45.1^+^) mice with OT-II or C57Bl/6 (CD45.2^+^) mice, respectively. *Ifng* ^-/-^ and *Ifnar1* ^-/-^ mice were crossed to OT-II B6xB6.SJL mice to generate *Ifng* ^-/-^ and *Ifnar1* ^-/-^ OT-II mice. KN2-OT-II mice were generated by crossing KN2 and OTII mice. *CD11c-Cre.Ifnar1^fl/fl^* and *XCR1-cre*.*Ifnar1^fl/fl^* mice were generated by crossing *Ifnar1^fl/fl^* to *CD11c-cre* and *XCR1-cre*, respectively. Mice were included in experiments at 8-12 weeks of age.

### Adoptive Transfers, Immunizations and mAb Treatment

CD4^+^ OT-II cells were isolated from spleen and LNs from OT-II*B6SJL mice with the EasySep mouse CD4^+^ T cell isolation kit (Stemcell Technologies, Vancouver, BC, Canada), according to manufacturer’s protocol. Enriched CD4^+^ cells (>90% purity) were labelled with 5μM CellTrace Violet (Life Technologies, Carlsbad, CA, USA) and 5000 - 5 x 10^5^ cells/recipient were transferred intravenously (i.v.) as indicated. 16-20 hours after transfer, recipients were immunized with 100 μg poly I:C (*In vivo*Gen) together with 300μg OVA or NP-OVA (Biosearch Technologies, Novato, CA, USA), as indicated, by intraperitoneal (i.p.) injection. Type I IFN and IFN-γ signaling was blocked by injection of 1 mg of anti-mIFNAR1 (MAR1-5A3) or anti-mIFN−γ (XMG1.2) respectively, at indicated time-points and control mice were treated with equal amounts of mIgG1 (MOPC-21) or rIgG1 (HRPN), all from BioXcell (West Lebanon, NH, USA).

### Bone Marrow Chimeras

To generate mixed BM chimeras, BM cells from age matched (8-12 weeks) wt, *Ifnar1^-/-^, Il27r^-/-^* or *Ifngr1^-/-^* donor mice were isolated and re-suspended in sterile PBS. A 1:1 mixture of wt and *Ifnar1^-/-^*, *Il27r^-/-^* or *Ifngr1^-/-^* BM cells (2-3x10^6^ total cells) were transferred into lethally irradiated (900 cGy) recipients (CD45.1^+^CD45.2^+^ B6.SJL x C57Bl/6 or CD45.1^+^B6.SJL). Recipient mice were thereafter kept on Ciprofloxacin for 2 weeks. At 8 weeks after transfer, mice were bled to assess reconstitution by flow cytometry. Whole BM chimeric mice were generated by reconstituting irradiated wt (C57Bl/6) and *Ifnar1^-/-^* mice with wt or *Ifnar1^-/-^* BM, otherwise as described above.

### Abs and Reagents

Flow-cytometry analyses were performed with Abs conjugated to FITC, PE, PerCP-Cy5.5, allophycocyanin, eFluor 450, Alexa Fluor 700, PE-Cy7, allophycocyanin-Cy7, Brilliant Violet 605, or biotin. The following Abs were used: anti-B220 (RA3-6B2), anti-CD4 (L3T4), anti-IFN-γ (XMG1.2), anti-GL-7 (GL-7), anti-CD38 (90), anti-T-bet (eBio4B10) (eBioscience, San Diego, CA, USA); anti-CXCR5 (2G8), anti-CD62L (MEL-14), anti-CD44, anti-CD95 (Jo2), anti-Bcl6 (K112-91), anti-TCR Vβ 5.1/5.2 (MR9-4), anti-TCR Vα2 (B20.1) (BD Biosciences, San Jose, CA, USA); anti-IgD (11-26c.2a), anti-CD138 (281–2), anti-CD45.1 (A20), anti-CD45.2 (104), anti-IgM (RMM-1), anti-IgG1 (RMG1-1), anti-IgG2b (RMG2b-1) (BioLegend, San Diego, CA, USA); anti-IgG2c (polyclonal) (Southern Biotech, Birmingham, AL, USA); and donkey anti-rat F(ab’)2 fragment (polyclonal) (Jackson Immunoresearch, West Grove, PA, USA). Streptavidin conjugated to eFluor450 (eBioscience), allophycocyanin (Biolegend), and PE (Southern Biotech) were used as secondary reagents in combination with biotinylated Abs. For detection of NP- or OVA-binding cells, PE-conjugated NP (Biosearch Technologies) or Alexa 647-conjugated OVA (Molecular Probes, Eugene, OR, USA) was used, respectively. Dead cells were excluded using propidium iodide or Live/Dead Fixable Aqua Dead Cell Stain Kit (Molecular Probes).

### Flow Cytometry

Single cell suspensions were prepared by mechanical disruption and filtered through 70µm cell strainers. RBCs were lysed with ACK buffer. For IgG analysis, cells were blocked with anti-FcR mAb (2.4G2) in 10% rat serum and thereafter incubated with isotype specific anti-IgG antibodies (see Abs and reagents). Remaining anti-mouse IgG reactivity were subsequently blocked with 10% mouse serum before incubation with fluorophore-conjugated mAbs. CXCR5 was detected as previously described (16) and followed by intracellular staining of Bcl6 and T-bet. Intracellular IFN-γ was detected after re-stimulation in complete medium with PMA (50 ng/ml) ionomycin (500 ng/ml; both Sigma-Aldrich, St. Louis, MN, USA), and Brefeldin A (eBioscience) for 3 hours. All intracellular staining was done using the FoxP3 Fixation/Permeabilization kit (eBioscience). Data was acquired on a LSRII or FACS AriaII and analyzed with FlowJo software (BD Bioscience).

### NP-Specific ELISA

NP-specific serum antibodies were measured by ELISA. 96-well EIA/RIA plates (Sigma Aldrich) were coated overnight with 0.5ug/ml NP_23_-BSA in PBS at room temperature and thereafter washed once in wash buffer (PBS+ 0.1% Tween20) and blocked with sample buffer (1% BSA in PBS) for 1 h and thereafter washed twice. Samples were diluted in sample buffer at 1:100 and 1:5000 and subsequently added in duplicates and incubated for 2 hours. Plates were washed four times before biotinylated anti-mouse IgG, anti-mouse IgG2c (polyclonal; both Southern Biotech), or anti-mouse IgG1 (RMG1-1) (BioLegend) was added in sample buffer, incubated for 1 h, and subsequently washed four times. Thereafter, Horseradish peroxidase (HRP) conjugated streptavidin was added and incubated for 45 minutes. After four washes, plates were developed with 3,3′,5,5′-Tetramethylbenzidine (TMB) and absorbance values were read at 450 nm on a SPECTROstar Nano (BMG Labtech, Ortenberg. Germany). NP-specific standard was prepared by pooling day 14 sera from NP-OVA immunized WT mice and used for all ELISAs to calculate serum titers (determined as 4x background OD value).

### Cell Sort and cDNA Preparation and Quantitative Real-Time PCR

F Total OT-II cells (Live, singlet, CD4^+^ CD45.1^+^ B220^-^) or GC B cells (Live, singlet, B220^+^Fas^+^CD38^-^ CD45.1^+^ or CD45.1^-^) were sorted from immunized recipients directly into RLT buffer supplemented with 1% β-ME (Qiagen, Hilden, Germany). mRNA was extracted with an RNeasy Micro Kit (Qiagen) and either used for sequencing or converted into cDNA using a SuperScript III First-Strand cDNA kit according to manufacturer’s protocol (Thermo Fisher, Waltham, MA, USA). Quantitative RT-PCRs were performed using SYBR GreenER qPCR SuperMix (mo Fisher) with 0.5 μM forward and reverse primers in a final volume of 20 μl. Reactions were run and recorded on an iCycler (Bio-Rad, Hercules, CA, USA).

### Primer Sequences

*Actb*: forward; 5′-CCACAGCTGAGAGGGAAATC-3′, reverse; 5′-CTTCTCCAGGGAGGAAGAGG-3′, *Bcl6*: forward; 5′-GTACCTGCAGATGGAGCATGT-3′; reverse; 5′-CTCTTCACGGGGAGGTTTAAGT-3′, *Tbx21*: forward; 5′-CAACAACCCCTTTGCCAAAG-3′; reverse; 5′-TCCCCCAAGCAGTTGACAGT-3′, *Ighg2c*: forward; 5′-CAGACCATCACCTGCAATGT-3′; reverse; 5′- CATGGGGGACACTCTTTGAG-3′, *Ctse*: forward; 5′-ATTCTGGAGGTCTCATAACTTGGAC-3′; reverse; 5′- TGCCAAAGTATTCCATATCCAGGTA-3′.

### Bulk mRNA Sequencing and Analysis

Paired-end RNA sequences were processed with AdapterRemoval (v. 2.1.3) (Lindgreen, 2012), setting the minimum quality to 20, minimum length to 25, collapsing reads when possible and removing Nextera adapters. The first seven base pairs were removed them using seqtk (v. 1.0). For each sample, files with collapsed and singleton reads were aligned to the mouse genome (Ensembl GRCm38.84) using HISAT2 (v. 2.0.1) (Kim et al., 2019). Gene expression profiles were generated using HTSeq (v. 0.11.1) (Anders et al., 2015) with default settings. For each group, genes that had >50 raw counts in >2 samples in either the wildtype- or the knockout condition were included in the analysis. Differential expression analyses were performed in R (R Core Team, 2018) using the edgeR package (McCarthy et al., 2012). Normalization factors were calculated using the trimmed mean of M-values (TMM) method. The edgeR general linear model was used for the differential expression analysis. Differentially expressed genes with a false discovery rate of <5% were considered statistically significant.

### Statistical Analysis

Data were analyzed with Prism version 6.0 (GraphPad Software). Analysis of statistical significance was done using one-way ANOVA with Kruskal-Wallis multiple comparison test for tree or more groups, or Mann-Whitney U test for two unpaired groups. Differences were considered significant when p ≤ 0.05 (*p≤0.05, **p<0.01and ***p<0.001).

## Results

### Reduced GC B Cell Response in Ifnar1^-/-^ Mice Involves Loss of Both IgG1^+^ and IgG2c^+^ GC B Cells

**Figure 1:**
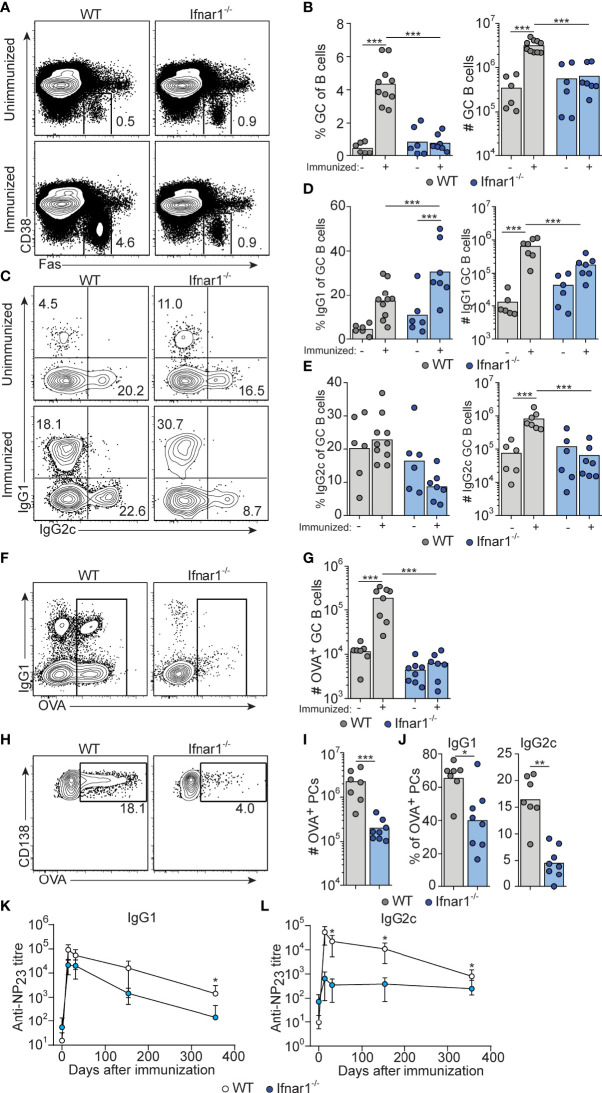
Attenuated GC B cell responses in *Ifnar1^-/-^* mice. WT or *Ifnar1* ^-/-^ mice were transferred with 5000 OTII cells and immunized with OVA/poly I:C **(A–J)** or NP-OVA/poly I:C **(K, L)**. **(A–J)** Flow cytometry analysis of splenocytes 8 days **(A–E** and **H–J)** or 14 days **(F, G)** after immunization. **(A, B)** Analysis of GC B cells after gating on total B cells (CD4^-^ B220^+^). Representative plots **(A)** and pooled results of percentages and numbers of GC B cells **(B)** are shown. **(C–E)** IgG subclass distribution among GC B cells. Representative contour plots of IgG1 versus IgG2c expression **(C)** and pooled results of percentages and numbers of IgG1^+^ **(D)** and IgG2c^+^ **(E)** GC B cells are shown. **(F, G)** OVA-binding GC B cells. Representative contour plots of binding of OVA-Alexa647 versus IgG1 expression after gating on GC B cells **(F)** and pooled results of numbers of OVA-binding GC B cells **(G)** are shown. **(H–J)** OVA-binding PCs. Representative contour plots showing intracellular binding of OVA-Alexa647 after gating on B220^low^ CD138^+^ PCs **(H)**, and pooled results of total number **(I)** and percentage of IgG1^+^ or IgG2c^+^ **(J)** OVA-binding PCs. Results are pooled from three **(A–E, H–J)** or two **(F, G)** individual experiments and symbols represents individual mice. **(K, L)** Serum titers of NP_23_-specific IgG1 **(K)** and IgG2c **(L)**, before and indicated time points after immunization. Results obtained from one experiment where mice (n=6 per group) were monitored over one year period of time. *p≤0.05, **p<0.01 and ***p<0.001.

To determine how type I IFNs affect GC B cell differentiation at the level of individual IgG subclasses, *Ifnar1* ^-/-^ mice were immunized i.p. with OVA and poly I:C. To visualize concurrent CD4 T cell responses (see below), mice received 5000 CD45 congenic OT-II cells. Thus, all cells are unable to respond to type I IFNs in these recipients except for a small and physiologically relevant number of antigen-specific CD4 T cells. The percentage and number of GC B cells were similar between unimmunized wt and *Ifnar1* ^-/-^ mice (**Figures 1A, B** and **Suppelementary Figure S1**), in line with reports of an important role for B cell IFN-γ receptor signaling, and not type I IFNs, in driving spontaneous GC formation in wt C57BL/6 mice (37, 38). When analyzed 3 days after immunization, neither wt nor *Ifnar1* ^-/-^ mice had mounted a GC B cell response exceeding the pre-existing GC B cell numbers (**Figure S1**). Eight days after immunization, a strong splenic GC B cell response had developed in wt mice (**Figures 1A, B**). Such response was however not evident in immunized *Ifnar1* ^-/-^ mice (**Figures 1A, B**). Furthermore, the absence of increased GC B cell numbers eight days after immunization in these mice was not merely due to delayed kinetics in the absence of type I IFN signaling, as numbers of GC B cells remained unchanged relative to unimmunized *Ifnar1* ^-/-^ controls 14 days after immunization (**Suppelementary Figure 1**).

The response induced by immunization of wt mice involved similar numbers of IgG1^+^ and IgG2c^+^ GC B cells (**Figures 1C–E** and **Suppelementary Figure S1**). Due to the robust magnitude of this response, pre-existing GCs made a very small and negligible contribution to the isotypes expressed by GC B cells in immunized wt mice. In *Ifnar1* ^-/-^ mice, we observed a significantly increased percentage of IgG1^+^ GC B cells after immunization (**Figure 1C, D** and **Suppelementary Figure S1**), indicating that an IgG1^+^ GC B cell response to some extent had developed also in the absence of type I IFN signaling. However, in this set of experiments we could not detect a corresponding increase in the number of IgG1^+^ GC B cells (**Figure 1D** and **Suppelementary Figure S1**). In contrast to IgG1, there was no significant increase in either percentage or number of IgG2c^+^ GC B cells in immunized as compared to non-immunized *Ifnar1* ^-/-^ mice (**Figures 1C, E** and **Suppelementary Figure S1**). Finally, the impairment of the antigen-specific GC B cell response in *Ifnar1* ^-/-^ mice was confirmed by OVA staining of GC B cells at day 14 (**Figures 1F, G**).

The majority of antigen-specific plasma cells (PCs) present in spleen around eight days after immunization are thought to be derived from GCs (39). The total number of splenic OVA-binding CD138^+^ PCs was approximately 10-fold lower in *Ifnar1^-/-^* compared to wt mice (**Figures 1H, I**), and both IgG1- and in particular IgG2c-producing cells were affected (**Figure 1J**). In addition, the few IgG1^+^ PCs present in *Ifnar1^-/-^* mice appeared to produce antibodies of lower affinity than their counterparts in wt animals, as indicated by a reduced percentage of OVA-binding IgG1 PC binding high levels of OVA in these mice (**Suppelementary Figure S1**). These results were further corroborated by serological studies where *Ifnar1* ^-/-^ mice essentially failed to mount an NP-specific IgG2c response after administration of NP-conjugated OVA plus poly I:C whereas their NP-specific IgG1 titers were reduced only 6.9- and 4.2-fold at two and four weeks after immunization, respectively (**Figure 1K, L**). Antigen-specific IgG1 titers however then declined more rapidly in the *Ifnar1* ^-/-^ mice and after one year they had an almost 80-fold lower NP-specific IgG1 titer than wt controls, indicating that the longevity of the specific IgG1 response was affected in the absence of type I IFN signaling (**Figure 1K**). Collectively these results demonstrate that type I IFNs are essential for GC formation induced by poly I:C, affecting both the quantity and quality of the response and, moreover, demonstrate that type I IFN signaling promotes the generation of both IgG1^+^ and, in particular, IgG2c^+^ GC B cells.

### Tfh Cells and IFN-γ Producing Th1 Cells Develop Concurrently in Response to Poly I:C and Are Both Reduced in *Ifnar1^-/-^* Mice

**Figure 2:**
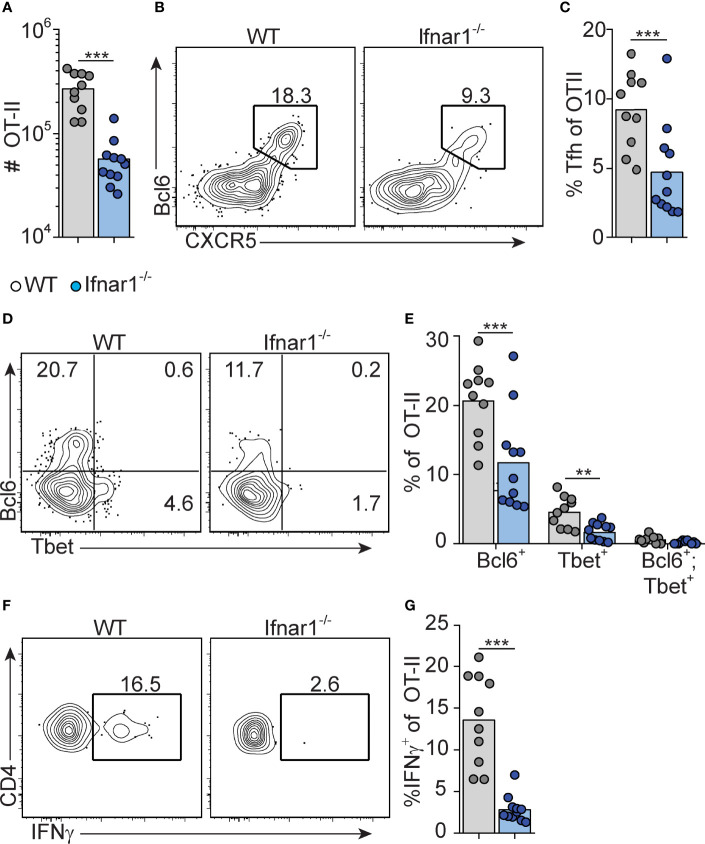
Type I IFN signaling augments Tfh and is required for Th1 cell differentiation. WT and *Ifnar1^-/-^* mice were transferred with 5000 OT-II cells and immunized with OVA/pI:C. Donor B220^-^ CD4^+^ CD45.2^+^ OT-II cells in spleens were analyzed by flow cytometry 8 dpi. **(A)** Number of OT-II cells. **(B, C)** Quantification of CXCR5^+^Bcl6^+^ Tfh cells. Representative contour plots **(B)** and pooled results of percentages of Tfh cells **(C)**. **(D, E)** Analysis of Bcl6 versus T-bet expression. Representative contour plots **(D)** and pooled results of percentages of OT-II cells displaying single- or co-expression of Bcl6 and T-bet, respectively. **(F, G)** IFN-γ expression. Representative contour plots **(F)** and pooled results of percentage of OT-II cells expressing IFN-γ **(G)**. Results are pooled from three individual experiments and each symbol represents one mouse. **p<0.01 and ***p<0.001.

We have previously shown that early Tfh cell fate commitment is reduced in *Ifnar1* ^-/-^ mice (as assessed three days after immunization) (16). To assess the relationship between Tfh and Th1 cell development, and to determine how type I IFN impacts on the accumulation of these subsets at the peak of the GC reaction eight days after immunization, we analyzed transferred OT-II cells in the same cohorts of mice as described above. *Ifnar1^-/-^* mice had fewer total OT-II cells in their spleens as compared to wt controls, confirming that type I IFNs enhance expansion and survival of activated CD4^+^ T cells (40, 41) (**Figure 2A**). Furthermore, Tfh cells were additionally affected in the *Ifnar1* ^-/-^ mice, as evident from a significantly reduced percentage of CXCR5^high^ Bcl6^+^ OT-II cells (**Figures 2B, C**). T-bet drives Th1 cell differentiation and is required for IFN-γ expression by CD4^+^ T cells (42). OT-II cells with detectable T-bet expression were confined to the Bcl6^-^ subset in the wt animals and in agreement with previously published results (31) IFN-γ expression was essentially lost in the *Ifnar1^-/-^* recipients, as was expression of T-bet (**Figures 2D–G**). Collectively, these results show that Tfh cells and IFN-γ producing Th1 cells exist as mutually exclusive subsets at the peak of the GC reaction and that both subsets depend on type I IFNs for their development.

### Early IFN-γ Derived From Cognate CD4+ T Cells Acts Directly on B Cells to Drive IgG2c CSR Without Enhancing the Overall Magnitude of the GC B Cell Response

**Figure 3:**
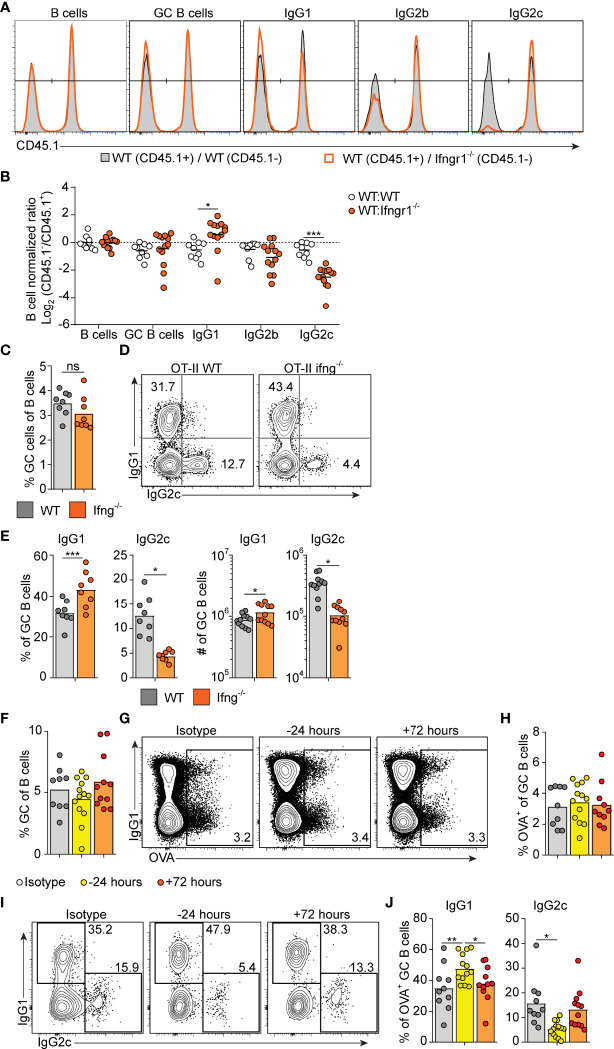
Early IFN-γ derived from cognate CD4 T cells acts on B cells to drive IgG2c CSR. **(A, B)** Mixed BM chimeras were generated by reconstituting WT recipients with a 1:1 mix of CD45.1^+^ CD45.2^+^ WT and CD45.1^-^ CD45.2^+^ WT or *Ifngr1^-^* ^/-^ BM cells. Eight - 10 weeks after reconstitution, chimeras were immunized with OVA/poly I:C and the proportion of CD45.1^+^ versus CD45.1^-^ cells among total splenic B cells and within indicated GC B cell population determined by flow cytometry 8 dpi. **(A)** Representative results for control (WT : WT) and experimental (WT : *Ifngr1^-^* ^/-^) BM chimeras. **(B)** Pooled results of log_2_ normalized CD45.1^-^ to CD45.1^+^ cell ratio in individual control and experimental chimeric animals. **(C–E)** *Ifng^-/-^* mice were transferred with 50 000 WT or *Ifng^-/-^* OT-II cells and immunized with OVA/poly I:C. Splenocytes were analyzed by flow cytometry 8 days later. **(C)** Percentage of GC B cells of total B cells. **(D, E)** IgG subclass expression by GC B cells. Representative contour plots **(D)** and pooled results of percentages and numbers of IgG1^+^ and IgG2^+^ GC B cells **(E)**. **(F–J)** WT mice were treated i.p. with 1 mg anti-IFN-γ mAb or isotype control 16 hrs before or 72 hrs after immunization with OVA/poly I:C. Mice were transferred with 50 000 OT-II cells 16 hrs before immunization and splenocytes were analyzed 8 dpi. **(F)** Percentage GC B cells of total B cells. **(G, H)** Analysis of OVA-binding by GC B cells. Representative flow cytometry results of OVA-Alexa647 fluorescence versus IgG1 expression **(G)** and pooled results of percentages of OVA^+^ GC B cells **(H)**. **(I–J)** IgG subclass expression by OVA^+^ GC B cells. Representative contour plots **(I)** and pooled results of percentages IgG1^+^ and IgG2^+^ GC B cells **(J)**. Pooled results from two **(C–E)** or three **(A, B, F–J)** experiments are shown. Each symbol represents one mouse. ns = not significant, *p≤0.05 and **p<0.01.

IFN-γ represents a well-established *in vitro* switch factor for the IgG2a/c subclass (43). B cell intrinsic IFN-γ signaling can also underlie GC formation in murine autoimmune models (37, 38). To determine the role of B cell intrinsic IFN-γ signaling in the GC B cell response driven by OVA/poly I:C, we made mixed bone marrow (BM) chimeric mice. Irradiated wt mice were grafted with wt BM (CD45.1^+^, CD45.2^+^) mixed with *Ifngr1* ^-/-^ or wt control BM (both CD45.2 single positive) at a 1:1 ratio. Analysis of splenocytes eight days after immunization showed that GC B cells developed equally well from *Ifngr1* ^-/-^ and wt B cells (**Figures 3A, B**), demonstrating that IFN-γ signaling in B cells is not involved in the type I IFN dependent GC B cell expansion. In contrast, CSR to IgG2c was strongly reduced in the *Ifngr1* ^-/-^ as compared to wt GC B cells present in the same mouse (**Figures 3A, B**). In agreement with the ability of IFN-γ to inhibit CSR to IgG1 and IgE (29, 43), the impaired IgG2c CSR in *Ifngr1* ^-/-^ B cells was compensated by an increased percentage of IgG1^+^ GC B cells (**Figures 3A, B**). Given that IL-27 represents an additional IgG2a/c switch factor (44) and can be produced by myeloid cells in response to type I IFNs (45, 46), we performed analogous experiments with mixed wt/*Il27ra* ^-/-^ BM chimeras. These experiments did not reveal any significant differences between wt and *Il27ra* ^-/-^ B cells (**Suppelementary Figures S2A, B**).

To determine if IFN-γ from cognate CD4^+^ T cells is sufficient for driving IgG2c CSR in B cells, we transferred IFN-γ sufficient or deficient OT-II cells into *Ifng^-/-^* mice and again evaluated the splenic response eight days after immunization. IFN-γ-deficient OT-II cells supported a GC B cell response of a comparable magnitude as their wt counterparts (**Figure 3C**). However, compared to mice receiving wt OT-II cells, recipients of *Ifng* ^-/-^ OT-II cells developed GCs with a strong and selective reduction in IgG2c, again compensated by an increase in IgG1 (**Figure 3D, E**). These experiments thus demonstrate an important role for IFN-γ derived from cognate CD4^+^ T cells in stimulating IgG2c CSR, but not for enhancing the overall magnitude of the GCs.

IFN-γ producing Tfh cells have been suggested to underpin IgG2a/c CSR within GCs (47). Given that Tfh cells largely lacked expression of T-bet at the peak of the poly I:C driven GC response (see **Figures 2D, E**), we considered the possibility that IFN-γ stimulates IgG2c CSR at an earlier stage and before evident GC formation. To assess this, we injected an IFN-γ neutralizing antibody either before or 72 hours after immunization. Regardless of the timing, this treatment had no impact on the percentage of total GC B cells detected eight days after immunization and staining with fluorescently labeled OVA confirmed an equal percentage of OVA-specific GC B cells in both experimental groups and in mice receiving an isotype control mAb (**Figures 3F–H**). However, treatment with anti-IFN-γ before immunization resulted in significantly fewer OVA-specific GC B cells expressing IgG2c as compared to isotype control treated mice (**Figure 3I, J**). This reduction was again reciprocated by an increased percentage of cells expressing IgG1 (**Figures 3I, J**). No such effects on the IgG subclass distribution were observed when treatment with anti-IFN-γ instead was started 72 hours after immunization (**Figures 3I, J**). Accordingly, IFN-γ acts on B cells within the first 72 hours after immunization and prior to GC formation to initiate the IgG2c CSR process and thereafter appears to be redundant for the IgG2c associated GC B cell response.

### B Cell Intrinsic Type I IFN Signaling Acts in Synergy With Type I IFN Dependent IFN-γ to Select the IgG2c Subclass

**Figure 4:**
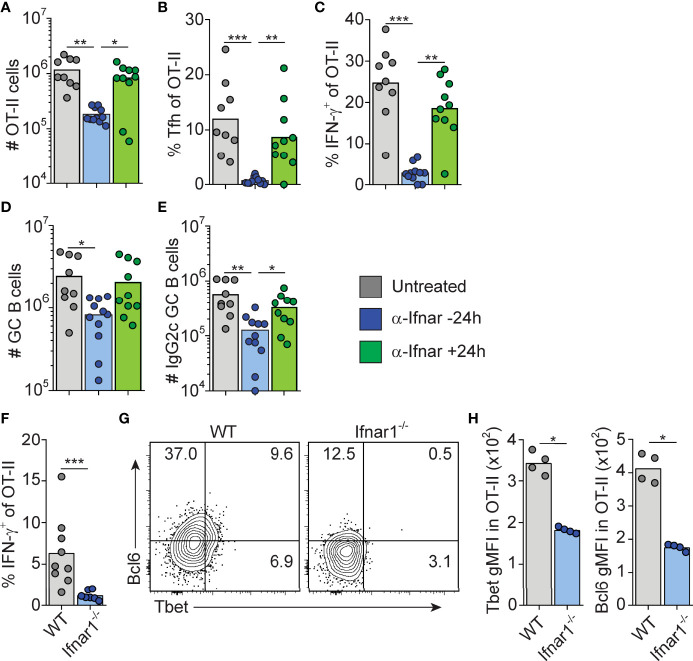
Type I IFN signaling drives Tfh and Th1 cell differentiation within the first 24 hrs after immunization. **(A–E)** WT mice transferred with 50000 OT-II cells were treated i.p. with 1 mg anti-IFNAR1 or isotype control mAb 16 hrs before or 24 hrs after immunization with OVA/pI:C. Splenocytes were analyzed by flow cytometry 8 dpi. **(A)** Number of OT-II cells. **(B)** Percentage of Tfh cells of total OT-II cells. **(C)** Percentage of IFN-γ^+^ cells of total OT-II cells. **(D)** Number of GC B cells. **(E)** Number of IgG2c^+^ GC B cells. **(F–H)** WT and *Ifnar1^-/-^* mice were transferred with 500 000 OT-II cells and donor cells in spleen analyzed by flow cytometry 3 days after immunization with OVA/poly I:C. **(F)** Percentage of IFN-γ^+^ cells of total OT-II cells. **(G–H)** Analysis of T-bet versus Bcl6 expression with representative contour plots **(G)** and geometric mean fluorescence intensity (GMFI) for T-bet and Bcl6, respectively. Individual mice and mean values **(A–F)** or representative results **(G–H)** of three independent experiments are shown. *p≤0.05, **p<0.01 and ***p<0.001.

The finding that B cell intrinsic IFN-γ signaling involved in IgG2c CSR precedes GC formation predicts that 1) type I IFN dependent priming of IFN-γ producing Th1 cells also occurs early in the response and 2) cognate IFN-γ producing CD4 T cells appear earlier than detectable GCs. To test the first prediction, we neutralized type I IFN signaling by injecting an anti-IFNAR1 mAb either 16 hours before or 24 hours after immunization. Neutralization of type I IFN signaling before, but not after, immunization inhibited OT-II cell expansion, Tfh cell differentiation and IFN-γ production as assessed eight days after immunization (**Figures 4A–C**). Consistent with the reduced number of Tfh cells, the overall magnitude of the GC B cell response was reduced when anti-IFNAR1 treatment preceded immunization (**Figure 4D**). Additionally, the numbers of IgG2c^+^ GC B cells were reduced after early but not late IFNAR1 blockade (**Figure 4E**). The type I IFN dependent signaling events that underlies Th1 development and IFN-γ dependent IgG2c CSR are therefore initiated during the first 24 hours of the poly I:C driven response.

To confirm that IFN-γ producing Th cells appear within the first 72 hours post-immunization, and to verify their dependence on upstream type I IFN signaling, we analyzed early Th cell differentiation in wt and *Ifnar1* ^-/-^ mice, respectively. IFN-γ producing OT-II cells were indeed detectable in wt mice 3 days after administration of OVA and poly I:C and the IFN-γ response was reduced in the *Ifnar1^-/-^* mice also at this earlier time point (**Figure 4F**). This correlated with significantly less T-bet protein in OT-II cells recovered from the *Ifnar1^-/-^* recipients (**Figures 4G, H**). Likewise, and as reported earlier (16), a reduction in OT-II cell Bcl6 expression was also evident already at this early stage in the *Ifnar1^-/-^* mice (**Figures 4G, H**). OT-II cells recovered from wt animals did however not express T-bet and Bcl6 in a mutually exclusive manner (**Figure 4G**), indicating the absence of clear Th1 versus Tfh cell dichotomy three days after immunization. Based on these results we conclude that type I IFNs drive development of the IFN-γ producing CD4 T cells that underpin IgG2c CSR within the first few days after immunization, prior to evident GC formation and appearance of fully committed Th1 and Tfh cells.

**Figure 5:**
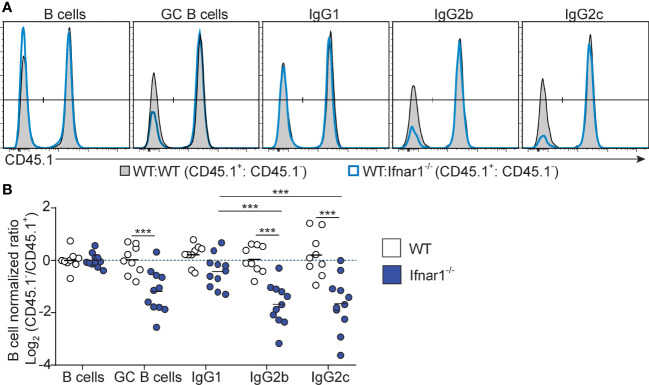
B cell intrinsic type I IFN signaling selectively amplifies the magnitude of IgG2^+^ GC B cell responses. Mixed BM chimeras were generated by reconstituting WT recipients with a 1:1 mix of CD45.1^+^ CD45.2^+^ WT and CD45.1^-^ CD45.2^+^ WT or *Ifnar1^-/-^* BM cells. Eight - 10 weeks after reconstitution, chimeras were immunized with OVA/poly I:C and the proportion of CD45.1^+^ versus CD45.1^-^ cells among total splenic B cells and within indicated GC B cell population determined by flow cytometry 8 dpi. **(A)** Representative results for control (WT : WT) and experimental (WT : *Ifnar1^-/-^*) BM chimeras. **(B)** Pooled results of log_2_ normalized CD45.1^-^ to CD45.1^+^ cell ratio in individual control and experimental chimeric animals. Results are from three individual experiments. Each symbol represents one mouse. *p≤0.05, **p<0.01 and ***p<0.001.

To determine how and to what extent direct type I IFN signaling in B cells influences the GC B cell response and IgG subclass selection after immunization with OVA/poly I:C, we again generated mixed BM chimeras, now by reconstituting irradiated wt mice with a 1:1 mix of wt with *Ifnar1^-/-^* or wt (control) BM. Analysis of immunized chimeric mice revealed that *Ifnar1* ^-/-^ B cells contributed to the GC B cell response to a lesser extent than IFNAR sufficient B cells present in the same animal. In this competitive setting, there was an approximately 2-fold lower number of *Ifnar1* ^-/-^ than wt GC B cells in the spleen (**Figure 5**). Strikingly, this reduction was mostly caused by a loss in IgG2c^+^ and IgG2b^+^ GC B cells; the frequency of GC B cells expressing either of these IgG subclasses was approximately 2-fold lower in the *Ifnar1* ^-/-^ than wt compartment of the same chimeric animal. Altogether, these results show that type I IFN augments the IgG2c^+^ and IgG2b^+^ GC B cell response through direct signaling in B cells and that, in contrast to IFN-γ, this effect goes beyond CSR and enhances the overall magnitude of the GC B cell response.

### Absence of B Cell Intrinsic Type I or Type II IFN Signaling Has Limited Effects on the Core GC B Cell Transcriptional Program but Results in Reduced T-Bet Expression and Altered Isotype Composition

Given the direct effect of both type I and type II IFN signaling, we next investigated how the respective IFN family influences the global transcriptional program of B cells within established GC. WT and IFN receptor deficient splenic GC B cells were thus sorted eight days after immunization from the same mixed BM chimeric mice described in **Figures 3**, **5**. Bulk mRNA sequencing analysis was preformed to generate datasets comparing gene expression in WT and *Ifnar1* ^-/-^ GC B cells or WT and *Ifngr1* ^-/-^ GC B cells, respectively.

To determine if IFN signaling had altered the core GC B cell program, we first compared these sequencing datasets to the transcriptional changes in GC B cell compared to naïve follicular B cells described in Shi et al. (48). We found that the GC B cell transcriptional signature was largely intact in the absence of either type I or type II IFN signaling (**Suppelementary Figures S3A, B**). Key GC B cell transcriptional changes, including mRNA encoding core transcription factors (*Bcl6* and *Bach2*), proteins involved in the somatic hypermutation program (*Aicda* and *Polh*), as well as proteins directing migration and localization of the B cells to the GC (*Sipr2*, *Gpr183*), were unaffected by the loss of B cell intrinsic IFN signaling. This suggests that type I IFN or IFN-γ signaling in B cells is not critical for the GC B cell program. We also compared our IFNAR dataset to the early type I IFN induced transcriptional changes in follicular B cells described by Mostavi et al. (49). Out of 71 genes that showed statistically significant differences in expression with at least a 2-fold change after IFN injection, only six were dysregulated in GC B cells at day eight after immunization with OVA and poly I:C (**Suppelementary Figure S3C**). This suggests that while a part of the IFN signature may be robustly conserved after IFN signaling has stopped, a majority of the IFN dependent transcriptional changes are transient.

**Figure 6:**
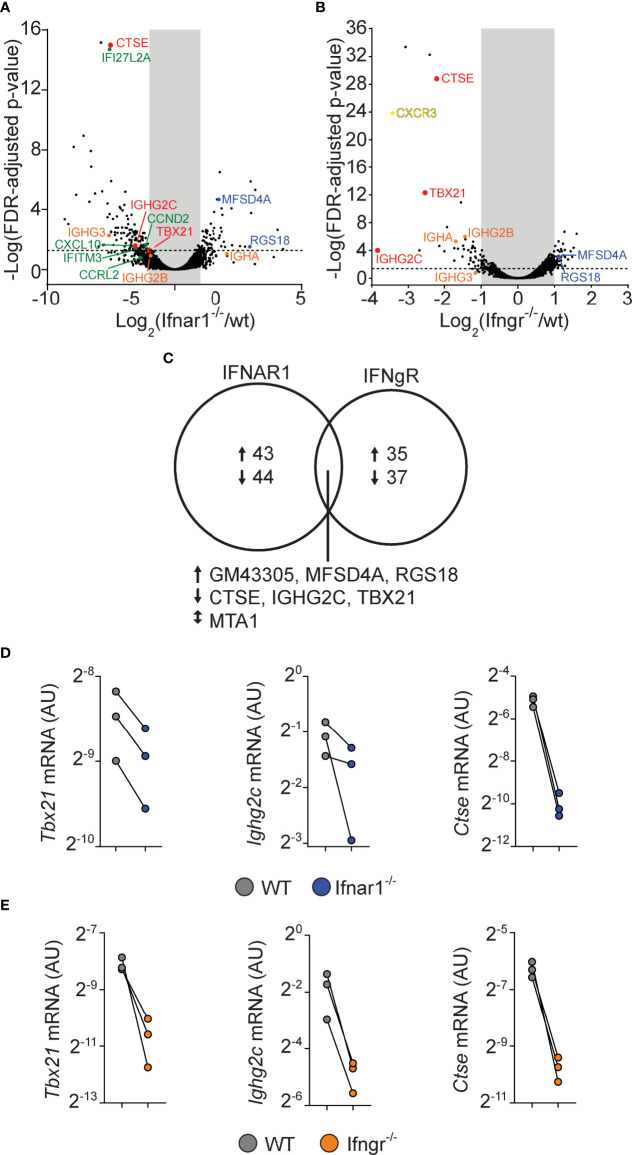
B cell intrinsic type I and type II signaling confers largely non-overlapping gene expression programs in GC B cells but co-regulate enhanced *Tbx21* and *Ighg2c* expression. WT and gene-targeted GC B cells were separately sorted from the WT : *Ifngr1^-^* ^/-^ and WT: *Ifnar1^-/-^* mixed BM chimeras described in figures 3 and 5, respectively, and processed gene expression analysis. **(A–C)** mRNA sequencing results. **(A, B)** Volcano plots of changes in gene expression between WT and *Ifnar1^-/-^* **(A)** or *Ifngr1^-/-^* **(B)** GC B cells. **(C)** Venn diagram showing all genes with >2-fold change and FDR < 0.05 when comparing WT and *Ifnar1^-/-^* or *Ifngr1^-^* ^/-^ GC B cells. The genes regulated by both type I and type II IFN signaling in GC B cells are listed below the diagram. **(D, E)** Quantitative rt-PCR analysis of gene expression in *Ifnar1^-/-^* **(D)** and *Ifngr1^-^* ^/-^ **(E)** GC B cells of genes identified by mRNA sequencing to rely on both type I and type II IFN B cell intrinsic signaling. Results are from three individual mice.

For both the IFNAR and IFNGR dataset, we filtered genes that displayed at least a 2-fold and statistically significant difference in expression between the WT and IFN receptor deficient cells (**Figures 6A, B**). By this approach, both cytokine families were found to influence a relatively limited number of genes, with only 87 and 72 genes being affected by type I IFN and IFN-γ signaling, respectively (**Figure 6C**). While the type I and type II IFN gene expression signatures have been difficult to separate when assaying peripheral blood during infections or in autoimmunity (50, 51), only seven genes were found to be dysregulated in both the IFNAR and IFNGR datasets. Of these, upregulation of three genes, (*Ctse, Tbx21, and Igh2c*) were dependent on both IFNAR and IFNGR signaling. The downregulation of these genes in IFN receptor deficient B cells was confirmed by qrt-PCR analysis from the same sorted GC B cell populations (**Figures 6D, E**). This suggests that B cell intrinsic type I IFN and IFN-γ signaling predominately affect different aspects of the GC B cell response but that the two pathways synergize to control class switching to IgG2c, likely through the induction of *Tbx21* (22). In addition to IgG2c, the differential gene expression identified in the two data sets indicated that both type I IFN and IFN-γ signaling in B cells affect the expression of other Ig isotypes. While both may have a small impact on IgG2b expression, we found type I IFN signaling to be associated with increased *Ighg3* mRNA expression. IFN-γ signaling was instead required for optimal *Igha* expression. Thus, while type I IFN and IFN-γ signaling collaboratively support switching to IgG2c, and possibly IgG2b, they may have unique roles in controlling switching to other isotypes.

### IFNAR on Non-Hematopoietic Cells and Cognate CD4 T Cells Is Dispensable for Th1 and Tfh Cell Differentiation After Poly I:C Adjuvanted Immunization

**Figure 7:**
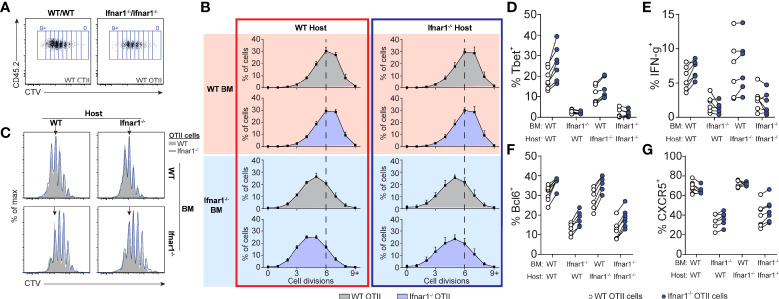
Tfh- and Th1-cell development is supported by IFNAR-signaling in hematopoietic cells distinct from cognate CD4 T cells. Equal numbers (250 000) of CTV-labelled WT and *Ifnar1^-/-^* OT-II cells were co-transferred into *Ifnar1^-/-^* BM chimeric recipient mice and spleens were analyzed 3 days after immunization with OVA/poly I:C. **(A)** Representative CTV dot plots showing gating strategy of OT-II cells from mice receiving either WT or *Ifnar1^-/-^* BM. **(B)** Representative CTV profiles of OT-II cells from all experimental groups. Arrows indicate median division in the full WT group. **(C)** Percentages (mean ± SD) of OT-II cells in indicated cell cycle number as determined by CTV-dilution. Verticle lines indicate median division in the full WT group. **(D–G)** Percentage of T-bet^+^ **(D)**, IFN-γ^+^ **(E)**, Bcl6^+^ **(F)**, and CXCR5^+^ **(G)** WT and *Ifnar1^-/-^* OT-II cells, respectively. Results are pooled from two individual experiments consisting of a total of 6-7 mice per group. Each pair of symbols represents one mouse **(D–G)**.

Given that type I IFNs are required for optimal generation of both Tfh and Th1 cells in poly I:C/OVA immunized mice, we next set out to determine the cellular targets for type I IFN signaling in the respective differentiation pathways. To this end we co-transferred CTV-labeled wt and *Ifnar1^-/-^* OT-II cells into BM chimeras, lacking IFNAR in either the hematopoietic, the non-hematopoietic (radio resistant) or both cell compartments. Cell cycle dependent dilution of CTV was examined three days after immunization (**Figures 7A–C**). The ability of type I IFNs to enhance CD4 T cell proliferation tracked with IFNAR expression by the BM donor cells (i.e. hematopoietic non T cell-intrinsic), likely reflecting the ability of type I IFNs to improve the antigen-presenting function and co-stimulatory capacity of APCs (16, 31, 52, 53). No change in CTV dilution was observed when comparing wt and *Ifnar1^-/-^* OT-II cells or wt and *Ifnar1^-/-^* irradiated recipient mice, demonstrating that T cell-intrinsic type I IFN signaling or signaling in radio resistant cells has no impact on early T cell proliferation under these conditions. The ability of type I IFNs to enhance Th1-associated T-bet and IFN-γ (**Figures 7D, E**) as well as Tfh cell associated Bcl6 and CXCR5 (**Figures 7F, G**) expression was also mostly a result of IFNAR expression on the BM donor cells. However, both T-bet and Bcl6 expression was slightly higher in *Ifnar1^-/-^* than wt OT-II cells, indicating that type I IFNs to some extent can counteract both Th1 and Tfh cell development through direct effects on T cells (**Figures 7D, F**). These effects were however subdominant to the enhanced T-bet and Bcl6 expression driven by T cell extrinsic type I IFN signaling in hematopoietic cells. In conclusion, these results indicate that IFNAR on cognate CD4 T cells or in the non-hematopoietic cell compartment is redundant for both Th1 and Tfh cell fate commitment and hence unlikely to have a major impact on the GC B cell response through these pathways.

### Type I IFN Signaling in cDCs Orchestrates IgG Subclass Specific GC B Cell Differentiation Through IL-4-Secreting Tfh and IFN-γ υ> Producing Th1 Cells

**Figure 8:**
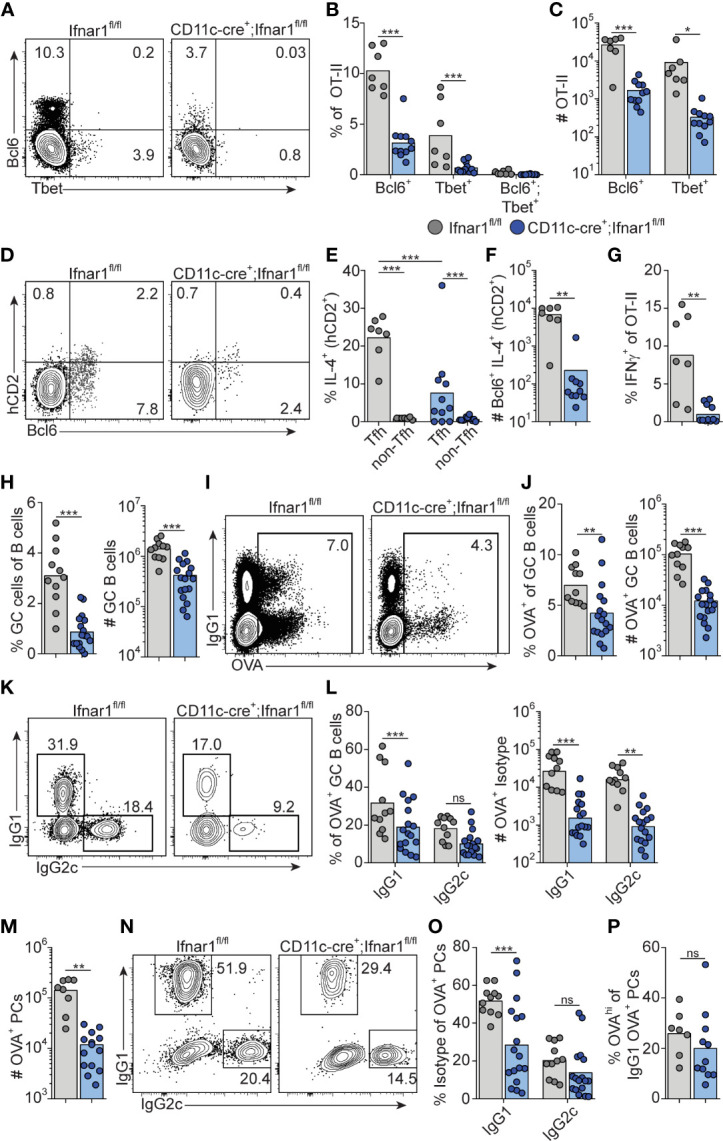
Type I IFN signaling in cDC regulates Th1, Tfh and GC B cell development. *Ifnar1^fl/fl^* and *CD11c-cre;Ifnar1^fl/fl^* mice were transferred with 50 000 KN2-OT-II cells, immunized with OVA/poly I:C and splenocytes were analyzed by flow cytometry 8 dpi. **(A–G)** Analysis of donor KN2-OT-II cells gated as B220^-^ CD4^+^ TCRVβ5.1^+^ TCRVα2^+^ CD44^+^ cells. **(A–C)** T-bet versus Bcl6 expression with representative contour plots **(A)** and pooled results of frequency **(B)** and number **(C)** of Bcl6^+^ and T-bet^+^ KN2-OT-II cells. **(D–F)** IL-4 secretion reported by hCD2 expression by Tfh (Bcl6^+^) donor cells. **(D)** Representative contour plots of Bcl6 versus hCD2 expression. **(E)** Percentage of IL-4 secreting Tfh and non-Tfh cells. **(F)** Number of IL-4 secreting Tfh cells. **(G)** Frequency of IFN-γ^+^ cells of total KN2-OT-II cells. **(H)** Percentages and number of GC B cells (B220^+^ CD95^+^ CD38^-^). **(I–L)** Analysis of OVA-Alexa647 binding GC B cells. **(I, J)** Representative contour plots of OVA-binding versus IgG1 expression **(I)** and pooled results of percentages and number of OVA^+^ GC B cells **(J)**. **(K, L)** Analysis of IgG subclass distribution by OVA^+^ GC B cells with representative contour plots of IgG2c versus IgG1 expression **(K)** and pooled results of percentages and numbers of IgG1^+^ and IgG2c^+^ OVA^+^ GC B cells **(L)**. **(M–P)** Analysis of OVA-Alexa647 binding PCs (B220^low^ CD138^+^). **(M)** Number of OVA^+^ PCs. **(N, O)** Analysis of IgG subclass distribution by OVA^+^ PCs with representative contour plots of IgG2c versus IgG1 expression (N) and pooled results of percentages of IgG1^+^ and IgG2c^+^ OVA^+^ PCs **(O)**. **(P)** Frequency of OVA^hi^ cells among OVA^+^ IgG1^+^ PCs. Results are pooled from three independent experiments. Each symbol represents one mouse. *p≤0.05, **p<0.01 and **p<0.001.

We have previously demonstrated a reduction in early Tfh cell differentiation in CD11c-cre.*Ifnar1* ^fl/fl^ mice with deletion of *Ifnar1* in cDCs (16). Although CD11c expression can be induced also in GC B cells approximately two days after immunization (54, 55), we found no alteration in early Tfh cell development in CD19-cre.*Ifnar1* ^fl/fl^ mice with specific deletion in B cells in our previous study (16). Combined with the results in the current study, showing that the poly I:C driven GC B cell response is influenced by type I IFN signaling only during the first 24 hours after immunization, this lead us to revisit the CD11c-cre.*Ifnar1* ^fl/fl^ model to determine how deletion of *Ifnar1* in cDCs impacts on the IgG subclass composition within GCs. In particular, while the results presented so far reveal how B cell intrinsic type I IFN signaling acts in synergy with the switch factor IFN-γ, produced from cognate CD4 T cells, to enhance IgG2c associated GC B cell responses, it was still unclear why IgG1^+^ GC B cells also are strongly reduced in the complete IFNAR knockout (see **Figure 1**). Selection of the IgG1 subclass is promoted by IL-4 (43) and within secondary lymphoid organs IL-4 secreting T cells are largely confined to the Tfh cell subset (47, 56). To visualize active secretion of IL-4 from cognate CD4 T cells, we intercrossed the OT-II strain with KN2 mice, reporting IL-4 secretion through expression of membrane anchored human CD2 (34). Similar to OT-II cells activated in wild type C57Bl/6 recipients (see **Figure 2D**), donor KN2-OT-II cells developed into mutually exclusive Th1 (T-bet^+^) and Tfh (Bcl6^+^) cell subsets eight days after immunization of *Ifnar1^fl/fl^* control mice (hereafter referred to as Cre^-^ control mice) while in *CD11c-Cre.Ifnar1^fl/fl^* mice both subsets were significantly reduced in frequency and number (**Figures 8A–C**). In the Cre^-^ control group, active IL-4 secretion was largely confined to the Bcl6^+^ Tfh cells and although their capacity to produce IL-4 appeared to be at least partially maintained in *CD11c-Cre.Ifnar1^fl/fl^* recipients (**Figures 8D, E**), the number of IL-4 secreting Tfh cell was dramatically reduced due to the overall weakened Tfh cell response in these mice (**Figure 8F**). Consistent with the impaired generation of T-bet^+^ Th1 cells, IFN-γ producing OT-II cells were also strongly diminished in *CD11c-Cre.Ifnar1^fl/fl^* mice (**Figure 8G**). Collectively, these results demonstrate a bifunctional effect of type I IFN signaling in cDCs to promote the appearance of separable IFN-γ producing Th1 and IL-4 producing Tfh cell subsets.

Similar to the complete *Ifnar1* knockout, the expansion of GC B cells was strongly reduced after immunization of *CD11c-Cre.Ifnar1^fl/fl^* mice compared with their Cre^-^ controls (**Figure 8H**). The requirement for IFNAR-expressing cDC was equally evident when analyzing the number of OVA-binding GC B cells in the two groups of mice, confirming that the reduction in GC B cells was related to the immunization rather than differences in pre-existing GC B cell numbers (**Figures 8I, J**). In marked contrast to when B cells specifically lack IFNAR, the reduced GC B cell response in the absence of IFNAR expressing cDCs was not caused by a selective loss of IgG2c expressing GC B cells; IgG1^+^ GC B cells were now at least equally affected, again confirmed by analysis of OVA-binding GC B cells (**Figures 8K, L**).

To determine if IFNAR signaling in cDC1 was required for any of the effects that type I IFNs have on Th1, Tfh and GC B cell differentiation, we immunized *XCR1-cre*.*Ifnar1^fl/fl^* mice (35) as above and examined the OT-II cell response (**Suppelementary Figures S4A, B**). No difference in OT-II cell expansion or differentiation to Th1 and Tfh cells was observed at day 8 post-immunization. Similarly, no effects on GC B cell expansion or generation of OVA-specific IgG1^+^ or IgG2c^+^ GC B cells were observed (**Suppelementary Figures S4C, D**).

Finally, we examined PC generation in immunized *CD11c-Cre.Ifnar1^fl/fl^* mice. Similar to the GC B cell response in these mice we found reduced numbers of OVA-specific PCs at day eight post-immunization (**Figure 8M**). While the total number of OVA-binding PC thus was more than 10-fold lower in *CD11c-Cre.Ifnar1^fl/fl^* mice as compared with Cre^-^ controls, the reduction was most pronounced for the IgG1 producing subset, as evident from a significant reduction in the percentage of IgG1^+^ but not IgG2c^+^ events when comparing the OVA-specific PC that had developed in the two mouse strains (**Figures 8N, O**). Of note, the reduced affinity of PCs developing in the complete IFNAR knockout was not apparent in the absence of IFNAR on cDCs, indicating that type I IFN signaling in B cells may underlie this effect (**Figure 8P**). Altogether these results show that expansion of IL-4 secreting Tfh cells, through type I IFN signaling in cDCs, represents a third pathway whereby type I IFNs regulate the GC response, providing an explanation to how type I IFN in addition to its pronounced effect on IgG2 also amplifies the IgG1 response.

## Discussion

Type I IFNs possess a wide range of immune stimulatory properties with relevance for antiviral immunity, vaccination, and systemic autoimmune disease. Here, we have dissected how a succinct type I IFN response induced by dsRNA drives GC formation and IgG subclass specification. By selectively blocking receptor signaling in defined target populations or permitting signaling to occur only during defined time windows, we demonstrate that type I IFNs primarily act on B cells and cDCs to rapidly program the B and T cell responses underlying formation of GCs and class switching to a broad IgG subclass distribution. In addition to enhancing generation of Tfh cells and long-lived IgG1 responses, type I IFNs induce T-bet expression and support IgG2c^+^ GC B cell development by mechanisms involving both direct effects on B cells and induction of IFN-γ production by cognate CD4 T cells.

Our results indicate that type I IFNs act on cDCs within the first 24 hours after immunization to initiate concurrent Tfh and Th1 cell differentiation. To assess the role of cDCs in this polarization process, we opted to delete *Ifnar1* in cDCs by using mice expressing the Cre recombinase under the control of the CD11c promoter. While CD11c also can be induced in GC B cells (54), which may have caused *Ifnar1* deletion also in GC B cells in the *CD11c-Cre.Ifnar1^fl/fl^* mice, this upregulation does not occur until two days after immunization (55). As we in the current study have isolated the effects of type I IFN to the first 24 hours after immunization, which is in line with the rapid and short-lived type I IFN response following poly I:C injections (31, 32), it is however unlikely that deletion of *Ifnar1* in GC B cells contributes to the observed phenotypes in this model. This conclusion is also in agreement with our previous study, showing a significant reduction in early Tfh cell development in *CD11c-Cre.Ifnar1^fl/fl^* mice but not in *CD19-Cre.Ifnar1^fl/fl^* mice with specific deletion in B cells (16).

Signaling in the cDC1 subset was redundant for both processes. It therefore seems likely that type I IFN signaling in cDC2 plays an important role for both Th cell fates, although we have not addressed this directly through cDC2 specific deletion of IFNAR. The demonstration that type I IFNs enhance both GC B cell responses and Th1 immunity stands in marked contrast to their immune suppressive role in chronic viral infections (4). The LCMV clone 13 strain establishes a persistent infection where long-lasting expression of IFN-β and IFN-α in cDCs leads to exhaustion of the protective Th1 cell response by a mechanism involving up-regulation of PD-L1 and IL-10 expression by the cDCs (57, 58). Likewise, the Th1-promoting property of cDC2 is suppressed by an excessive type I IFN response during severe blood-stage *Plasmodium* infection (59). The stimulatory effects of type I IFNs described in the current study are hence likely related to the short duration of the type I IFN response induced by poly I:C, a notion further supported by the beneficial effects these cytokines have in acute viral infections, including vesicular stomatitis virus (VSV) (19, 60), influenza (18), RSV (61) and adenovirus (13). Still, the ability of type I IFNs to concomitantly drive Th1 and Tfh cell development is not necessarily recapitulated in the acute viral infection models. While VSV similar to poly I:C gives rise to a rapid and transient peak of type I IFNs that stimulates generation of Tfh cells, this occurs at the expense of Th1 cell polarization (17). One explanation for this apparent discrepancy could be the mixed pro-inflammatory response induced by viral sensing through multiple innate receptors (62). The adjuvant effect of poly I:C is on the other hand completely lost in IFNAR deficient mice, as shown herein, and injection of purified IFN-β replicates the immune stimulatory effects of poly I:C when co-injected with a protein antigen (25).

The induction of T-bet associated GCs and long-lived IgG responses through the isolated effect of type I IFNs should be of considerable interest for the development of adjuvants to enhance antiviral vaccine efficacy. IgG2a/c represents the dominant anti-viral IgG subclass in mice (63) and B cell-specific T-bet deletion leads to impaired antiviral IgG2a/c production and viral clearance (64), as well as an inability to contain chronic viral infection (65). In addition, protective antibody responses against influenza were recently demonstrated to rely on T-bet associated GCs (24). The current study may also contribute towards understanding the immunogenicity of emerging mRNA vaccine approaches (66). Similar to poly I:C, mRNA vaccines induce a short-lived type I IFN response (33), and currently approved SARS-CoV-2 mRNA vaccines induce specific IgG serum concentrations higher or equivalent to the levels detected in convalescent sera (67, 68).

While antiviral immunity frequently has been associated with both type I IFN and IgG2a/c antibody production, it has not been clear how type I IFNs promote IgG2a/c dominated GCs. B cell-intrinsic type I IFN signaling was recently shown to be crucial for spontaneous development of IgG2c^+^ GCs in lupus prone *B6.Sle1b* mice (15). GC formation was however not affected by lack of type I IFN signaling when the same lupus-prone strain or wt mice were immunized with NP-conjugated chicken γ-globulin (15), probably reflecting an insufficient type I IFN response to this particular immunization regimen. In the current study, we show that IFN-γ production from cognate CD4 T cells, which requires type I IFN signaling in cDCs, is a critical component of the type I IFN-dependent IgG2c^+^ GC B cell response. This pathway was however not sufficient but acted in concert with direct sensing of type I IFNs by the B cells and both pathways contributed to the induction of T-bet expression in GC B cells. Yet, the effects of type I IFNs and IFN-γ differed. While IFN-γ acted as a non-redundant IgG2c switch factor, only signaling through IFNAR amplified GC B cell expansion with specific effects on IgG2c^+^ and IgG2b^+^ GC B cells. How this occurs and why the effect was confined to GC B cells expressing the IgG2 subclasses remains to be determined. However only early, and not late, IFNAR neutralization resulted in reduced IgG2c^+^ GC B cell numbers. Likewise, only few of the roughly 70 genes previously shown to be induced in B cells following type I IFN treatment (49) were affected in IFNAR deficient GC B cells eight days after immunization, further supporting that type I IFNs acted on the B cells early in the response, possibly during initial B cell activation. Indeed, type I IFNs have been shown to confer increased sensitivity to BCR stimulation and to promote B cell expansion by both enhancing proliferation and reducing sensitivity to apoptosis (15, 69).

Whereas targeted deletion of IFNγR in B cells, IFN-γ in cognate CD4 T cells or IFNAR in cDCs resulted in similar reduction in IgG2c^+^ GC B cells, IFNAR deletion in cDCs additionally impaired development of IgG1^+^ GC B cells and resulted in an overall reduced magnitude of the GC response. This coincided with reduced Tfh cell development, with a particularly pronounced effect on IL-4 producing Tfh cells. In the wt setting, Tfh cells thus became IL-4 producers also under the strong Th1 polarizing conditions otherwise conferred by poly I:C and downstream type I IFN production. In addition, T-bet was absent from the Tfh cells around the peak of the GC reaction, indicating limited Th1 cell characteristics of the Tfh cell subset. Consistent with this, we demonstrate that both IFN-γ and type I IFNs acted on B cells very early after immunization and induced IgG2c CSR prior to evident GC formation. These results are in agreement with previous studies, demonstrating that CSR mostly precedes GC formation (70–72). Nonetheless, similar to viral infection models (73–75), Bcl6 and T-bet were co-expressed by the T cells at this early stage. We could hence not identify divergent Th1 versus Tfh cell commitment at the time when B cells were receiving the IgG2c switch signals, in line with previous studies demonstrating incomplete commitment of the Bcl6-expressing Th cell subset at early stages of the GC response (76–78) Tfh cells with a history of T-bet expression have been shown to produce IFN-γ within established GCs (79). While it remains possible that equivalent IFN-γ producing T-bet^-^ Tfh cells were present within the GCs studied herein, late IFN-γ neutralization had no detectable effect on the magnitude or IgG subclass composition of the GC response. This indicates that the IgG2c^+^ GC B cells did not rely on continuous IFN-γ signaling within the GCs.

In summary, the current study describes how type I IFNs through at least three separate pathways can enhance and modulate the GC B cell response. Our results provide a detailed roadmap of how this family of cytokines confers long-lived humoral immunity. Exploiting the type I IFN dependent pathways identified herein could provide a means to enhance efficacy of e.g. mRNA vaccine regimens and to prolong the duration of vaccine-induced protection. On the other hand, the relative contribution of these pathways to onset of systemic autoimmune disease warrants further investigations.

## Data Availability Statement

The datasets presented in this study can be found in online repositories. The names of the repository/repositories and accession number(s) can be found below: https://www.ncbi.nlm.nih.gov/geo/, GSE201551.

## Ethics Statement

The animal study was reviewed and approved by Lund/Malmo animal ethical committee (Sweden).

## Author Contributions

BJ-L, MD and AP conceived of the study and designed experiments. MD and AP performed experiments and analyzed results. KN and SB analyzed RNA-seq data. BJ-L secured funding for the study. SB and KL provided essential resources. BJL supervised the project. MD, AP and BJ-L synthesized results and wrote the manuscript with input from co-authors. All authors contributed to the article and approved the submitted version.

## Funding

This work was supported by the Swedish Cancer foundation (Cancerfonden; 18 0324) and the Lundbeck foundation (R155-2014-4184). KL was supported by a Lundbeck Foundation Research Fellowship (R215-2015-4100). KN and SB were supported by the Novo Nordisk Foundation (NNF14CC0001).

## Conflict of Interest

The authors declare that the research was conducted in the absence of any commercial or financial relationships that could be construed as a potential conflict of interest.

## Publisher’s Note

All claims expressed in this article are solely those of the authors and do not necessarily represent those of their affiliated organizations, or those of the publisher, the editors and the reviewers. Any product that may be evaluated in this article, or claim that may be made by its manufacturer, is not guaranteed or endorsed by the publisher.

## Supplementary Material

Supplementary Figure 1 B cell responses in *Ifnar1* ^-/-^ mice. OTII cells were transferred into *Ifnar1* ^-/-^ and WT mice before immunization with OVA/poly I:C. Splenocytes were analyzed by flow cytometry three or 14 days later. **(A)** Number of GC B cells. **(B)** Number of IgG1^+^ GC B cells. **(C)** Number of IgG2c^+^ GC B cells. **(D-E)** Analysis of relative affinities of OVA-specific IgG1^+^ PCs. Representative contour plots **(D)** and pooled results showing frequency of OVA^hi^ PCs among total OVA^+^ IgG1^+^ PC eight days post infection **(E)**. Results are pooled from three **(A-C)** or two **(D-E)** independent experiments.

Supplementary Figure 2 IL-27R signaling in B cells is redundant for GC B cell responses. **(A, B)** Mixed chimeras were generated by reconstituting lethally irradiated WT (CD45.1^+^, CD45.2^+^) recipients with a 1:1 mix of WT (CD45.1^+^, CD45.2^+^) and WT or *Il27r* ^-/-^ (CD45.1^-^, CD45.2^+^) BM cells. 8-10 weeks after reconstitution, chimeras were immunized with OVA plus poly I:C, and splenic GC B cell responses were analyzed 8 days later. **(A)** Representative histograms of WT : WT (shaded) and WT : *Il27r^-/-^* chimeras (blue) showing the distribution of B cells, GC B cells and GC B cells expressing indicated IgG isotypes (IgG1^+^, IgG2b^+^ and IgG2c^+^). **(B)** Log_2_ normalized ratio of B cells, GC B cells and GC B cells expressing indicated IgG isotype (IgG1, IgG2b and IgG2c) in WT : WT and WT : *Il27r^-/-^* chimeras. Results are pooled from two **(A, B)** individual experiments, each symbol represents one mouse.

Supplementary Figure 3 The core GC B cell program is largely intact in absence of B cell intrinsic type I IFN and IFN-γ signaling. **(A, B)** RNA sequencing data from GC B cells with either IFNAR **(A)** or IFNγR **(B)** disruption was compared to sequencing data from Shi et al. (reference # 48), by plotting fold-change in GC B to naïve B cells vs *Ifnar1* ^-/-^ **(A)** or Ifngr^-/-^ **(B)** to WT cells. **(C)** RNA sequencing data from GC B cells with IFNAR disruption was compared to gene expression changes induced in B cells following administration of type I IFN to mice as described in Mostavi et al. (reference # 49). Genes induced by injection of type I IFN with >2-fold change induction and statistical significance were plotted against the fold change in *Ifnar1* ^-/-^ vs WT cells. Results are from three individual mice.

Supplementary Figure 4 Type 1 IFN signaling in cDC1 does not regulate Th1, Tfh or GC B cell responses. *Ifnar1^fl/fl^* and *XCR1-cre;Ifnar1^fl/fl^* mice were transferred with 50 000 OT-II cells and immunized with OVA/poly I:C. Lymphocyte responses in the spleen were analyzed 8 days later. **(A)** Number of OT-II cells from *Ifnar1^fl/fl^* and *XCR1-cre;Ifnar1^fl/fl^* mice. **(B)** Frequency of Bcl6^+^ and T-bet^+^ cells among transferred OT-II. **(C)** Number of total GC B cell. **(D)** Number of OVA^+^ GC B cell. Results are pooled from two independent experiments. Each symbol represents one mouse.
