# Supplementary figures and images for: Type I Interferons Promote Germinal Centers Through B Cell Intrinsic Signaling and Dendritic Cell Dependent Th1 and Tfh Cell Lineages
Source: Front Immunol. 2022 Jul 13;13:932388. doi: 10.3389/fimmu.2022.932388 (PMC9326081; doi:10.3389/fimmu.2022.932388)

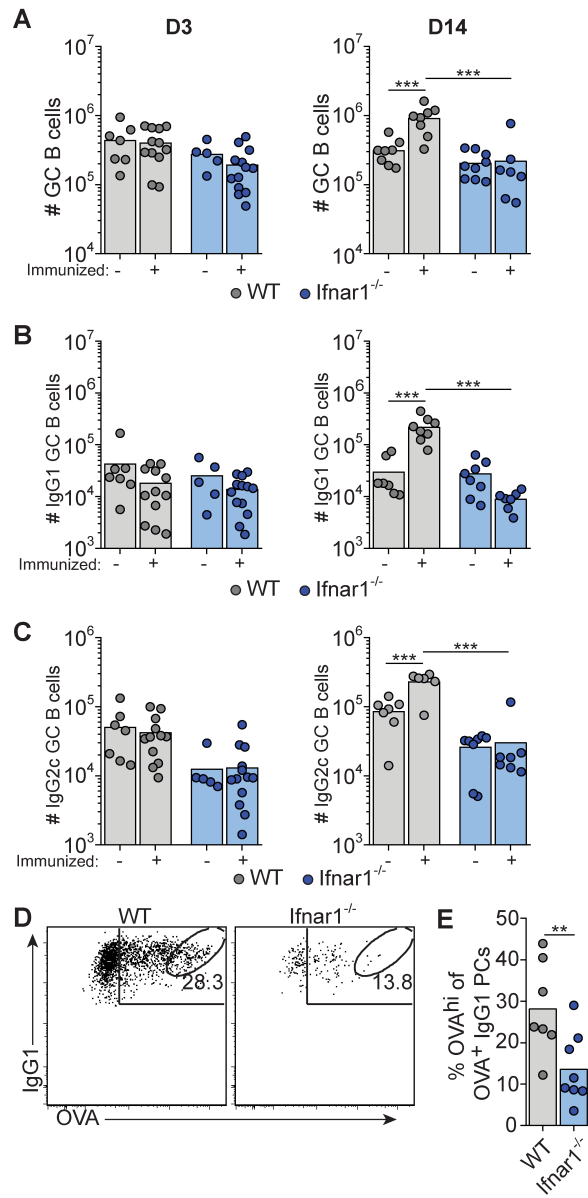

Supplement: Supplementary Figure 1 — B cell responses in Ifnar1 -/- mice. OTII cells were transferred into Ifnar1 -/- and WT mice before immunization with OVA/poly I:C. Splenocytes were analyzed by flow cytometry three or 14 days later. (A) Number of GC B cells. (B) Number of IgG1+ GC B cells. (C) Number of IgG2c+ GC B cells. (D-E) Analysis of relative affinities of OVA-specific IgG1+ PCs. Representative contour plots (D) and pooled results showing frequency of OVAhi PCs among total OVA+ IgG1+ PC eight days post infection (E). Results are pooled from three (A-C) or two (D-E) independent experiments. [file Image_1.pdf]

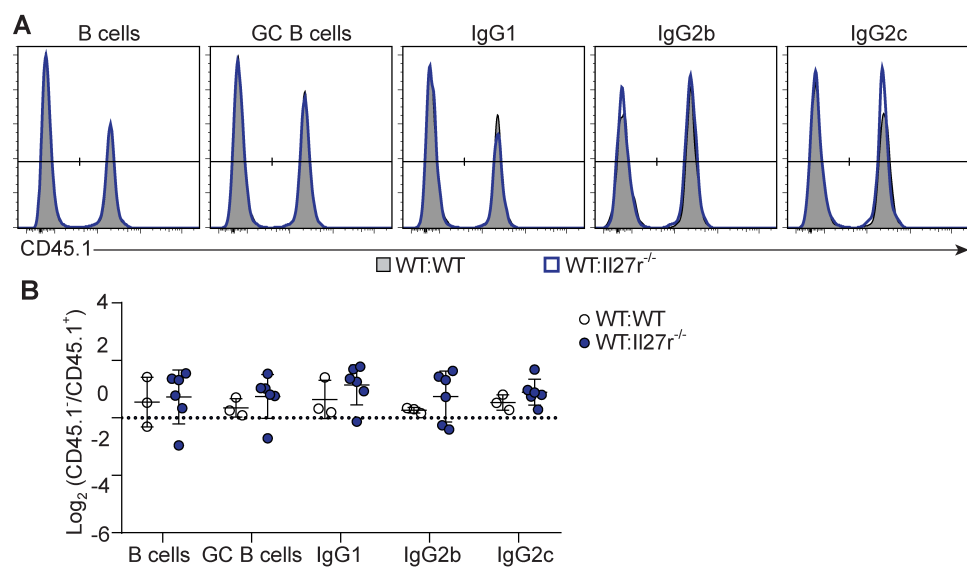

Supplement: Supplementary Figure 2 — IL-27R signaling in B cells is redundant for GC B cell responses. (A, B) Mixed chimeras were generated by reconstituting lethally irradiated WT (CD45.1+, CD45.2+) recipients with a 1:1 mix of WT (CD45.1+, CD45.2+) and WT or Il27r -/- (CD45.1-, CD45.2+) BM cells. 8-10 weeks after reconstitution, chimeras were immunized with OVA plus poly I:C, and splenic GC B cell responses were analyzed 8 days later. (A) Representative histograms of WT : WT (shaded) and WT : Il27r-/- chimeras (blue) showing the distribution of B cells, GC B cells and GC B cells expressing indicated IgG isotypes (IgG1+, IgG2b+ and IgG2c+). (B) Log2 normalized ratio of B cells, GC B cells and GC B cells expressing indicated IgG isotype (IgG1, IgG2b and IgG2c) in WT : WT and WT : Il27r-/- chimeras. Results are pooled from two (A, B) individual experiments, each symbol represents one mouse. [file Image_2.pdf]

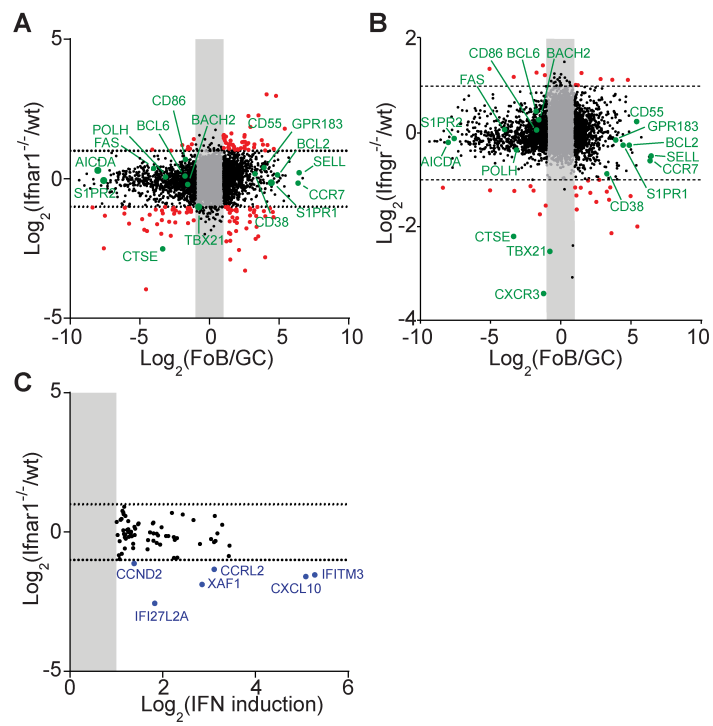

Supplement: Supplementary Figure 3 — The core GC B cell program is largely intact in absence of B cell intrinsic type I IFN and IFN-γ signaling. (A, B) RNA sequencing data from GC B cells with either IFNAR (A) or IFNγR (B) disruption was compared to sequencing data from Shi et al. (reference # 48), by plotting fold-change in GC B to naïve B cells vs Ifnar1 -/- (A) or Ifngr-/- (B) to WT cells. (C) RNA sequencing data from GC B cells with IFNAR disruption was compared to gene expression changes induced in B cells following administration of type I IFN to mice as described in Mostavi et al. (reference # 49). Genes induced by injection of type I IFN with >2-fold change induction and statistical significance were plotted against the fold change in Ifnar1 -/- vs WT cells. Results are from three individual mice. [file Image_3.pdf]

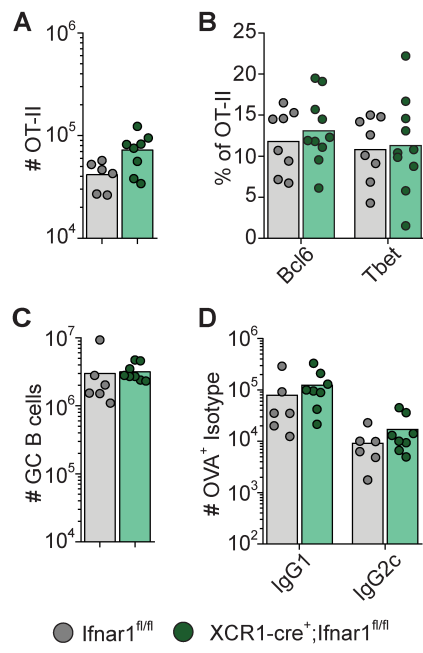

Supplement: Supplementary Figure 4 — Type 1 IFN signaling in cDC1 does not regulate Th1, Tfh or GC B cell responses. Ifnar1fl/fl and XCR1-cre;Ifnar1fl/fl mice were transferred with 50 000 OT-II cells and immunized with OVA/poly I:C. Lymphocyte responses in the spleen were analyzed 8 days later. (A) Number of OT-II cells from Ifnar1fl/fl and XCR1-cre;Ifnar1fl/fl mice. (B) Frequency of Bcl6+ and T-bet+ cells among transferred OT-II. (C) Number of total GC B cell. (D) Number of OVA+ GC B cell. Results are pooled from two independent experiments. Each symbol represents one mouse. [file Image_4.pdf]
